# Impact of dietary *Spirulina* on performance, antioxidant status, carcass traits and pathological alteration in broilers exposed to ochratoxin A stress

**DOI:** 10.3389/fvets.2024.1532353

**Published:** 2025-01-29

**Authors:** Ayman S. Salah, Mahmoud S. El-Tarabany, Marwa Mostafa, Rania Samir Zaki, Mahmoud M. Azzam, Omnia I. El Euony, Mahmoud Alagawany, Antonia Lestingi, Ahmed A. Elolimy, Mahmoud Madkour, Ahmed Fotouh

**Affiliations:** ^1^Department of Animal Nutrition and Clinical Nutrition, Faculty of Veterinary Medicine, New Valley University, El-Kharga, Egypt; ^2^Department of Animal Wealth Development, Faculty of Veterinary Medicine, Zagazig University, Sharkia, Egypt; ^3^Department of Pathology, Faculty of Veterinary Medicine, Benha University, Toukh, Egypt; ^4^Department of Food Hygiene, Safety and Technology, Faculty of Veterinary Medicine, New Valley University, El Kharga, Egypt; ^5^Department of Animal Production, College of Food and Agricultural Sciences, King Saud University, Riyadh, Saudi Arabia; ^6^Department of Forensic Medicine and Toxicology, Faculty of Veterinary Medicine, Alexandria University, Alexandria, Egypt; ^7^Poultry Department, Faculty of Agriculture, Zagazig University, Zagazig, Egypt; ^8^Department of Veterinary Medicine, University of Bari Aldo Moro, Valenzano, Italy; ^9^Department of Integrative Agriculture, College of Agriculture and Veterinary Medicine, United Arab Emirates University, Abu Dhabi, United Arab Emirates; ^10^Animal Production Department, National Research Centre, Giza, Egypt; ^11^Department of Pathology and Clinical Pathology, Faculty of Veterinary Medicine, New Valley University, El Kharga, Egypt

**Keywords:** broilers, OTA, *Spirulina platensis*, amelioration, mitigation, growth, blood

## Abstract

**Introduction:**

This study examined the influence of *Spirulina platensis*, ochratoxin A (OTA), and their combination on growth, antioxidant status, liver and kidney functions, immunity, and carcass traits of broiler chickens.

**Methods:**

160 unsexed 1-day broiler chicks were divided into four treatment groups, each consisting of 4 replications of 10 chicks. The duration of this study was six weeks, during which the experimental groups were organized as follows: G1 consumed a basal diet (control), G2 consumed a basal diet treated with OTA at a level of 1 mg/kg of diet, G3 consumed a basal diet treated with *Spirulina platensis* at a level of 1 g/kg of diet, G4 consumed a basal diet treated with OTA at a level of 1 mg/kg of diet and *Spirulina platensis* at a level of 1 g/kg diet.

**Results and discussion:**

The results illustrated that OTA-contaminated feed resulted in a significant elevation in total cholesterol, triglyceride, low- and very low density lipoprotein, and malondialdehyde, along with a significant reduction in antioxidant status and immunological response. On the other hand, *Spirulina* supplementation significantly enhanced performance performance (body weight, body weight gain and feed conversion ratio). Lipid profile was significantly decreased by *Spirulina* supplementation. Antioxidant activity (superoxide dismutase, catalase, total antioxidant capacity, and glutathione peroxidase) of broilers exposed to OTA was significantly increased by *Spirulina* supplementation. Finally, supplementing *Spirulina platensis* in broiler chickens fed on OTA contaminated diet attenuated the harmful effects of OTA, while improving the growth performance, antioxidant activity, lipid profile, and immune response of broiler chickens.

## Introduction

The prevalence of mycotoxins is a general problem that has negative effects on animals and humans (1). Several researchers have established that ochratoxin A (OTA) is one of the mycotoxins in the livestock and agriculture sectors. OTA is a mycotoxin commonly found in feed ingredients, particularly grains, and harms poultry health and productivity (1). Several research works have provided evidence of the beneficial effects of nutritional supplements with vitamins and electrolytes (2–6), probiotics, prebiotics (7–10), phytogenics and organic acids (11–13). Additionally, various feed additives and management practices are also included (14–18) in decreasing the detrimental effects of environmental stress on broiler chicken performance. The impact of *Spirulina platensis* supplementation on various physiological characteristics in broilers exposed to ochratoxin A (OTA) stress is a topic of significant interest within the realm of animal nutrition and health (19). Multiple research investigations have reported the influence of *Spirulina platensis* supplementation on broiler chickens under different stressors and conditions (20, 21). Mirzaie et al. (21) explored the influence of dietary supplementation with *Spirulina* on the immune system, lipid profile, antioxidant status, and performance traits of broiler chickens raised in hot environments, proposing that the addition of *Spirulina* could relieve the negative effects produced by elevated environmental temperature on a biochemical level. Furthermore, studies have shown that dietary supplementation of *Spirulina platensis* can benefit animal health (22, 23). For instance, Fries-Craft et al. (20) demonstrated that algae-based feed ingredients can protect the gastrointestinal tract health and modulate the immunity responses in poultry. Additionally, Eldesoky et al. (24) found that *Spirulina platensis* could improve testis injuries and sperm quality in rats exposed to mercuric chloride. Research by Park et al. (23) has demonstrated that including 1.0% *Spirulina* powder in the diet can be an alternative to enhance broiler chicken production. Studies by Qureshi et al. (25) have also shown the positive influence of *Spirulina* supplementation on the immune system, antioxidant status, and antibody formation in broiler chickens. Further, El-Shall et al. (26) have emphasized the ability of *Spirulina platensis* as a poultry feed supplement to enhance immunological regulation and growth performance, particularly in high ambient temperature environments. Additionally, Elbaz et al. (27) emphasized the beneficial effects of *Spirulina platensis* in mitigating the detrimental impacts due to heat stress on broiler chickens. Furthermore, Khalilnia et al. (28) indicated that including *Spirulina platensis* microalgae in broiler diets can lead to positive immune responses by increasing serum levels of immunoglobulins and phagocytic activity. Understanding the complex relationship between dietary *Spirulina platensis* supplementation and OTA-induced stress in broilers requires a thorough investigation covering various aspects of avian physiology. Key indicators of the broiler’s ability to utilize dietary nutrients and combat stressors include parameters such as feed consumption, body weight gain, and feed conversion ratio (29). The assessment of antioxidant status offers insights into cellular defense mechanisms against oxidative damage induced by OTA, with biomarkers such as superoxide dismutase (SOD) level, the activity of catalase (CAT), and glutathione peroxidase (GPx) activity reflecting antioxidant capacity. Liver and kidney function tests provide valuable information on the hepatic and renal health of broilers, which may be compromised under OTA-induced toxicity (21, 30). Evaluation of carcass traits, including carcass yield, meat quality attributes, and organ weights, reflects the overall performance and marketability of broiler chickens (31). Research has shown that OTA exerts its harmful effects by suppressing mitochondrial function, causing increased oxidative stress, and hindering protein synthesis.

By examining the impact of dietary *Spirulina platensis* supplementation on a wide range of physiological parameters in broilers exposed to OTA stress, researchers aim to uncover the potential mechanisms behind its protective effects and optimize its inclusion levels in poultry diets (21). It is hypothesized that the dietary addition of microalgae such as *Spirulina platensis* is expected to benefit poultry. The beneficial impacts of *Spirulina platensis* as a feed supplement has been previously studied separately and in the absence of natural pollutants such as OTA. Therefore, this study attempted to investigate the impact of *Spirulina platensis* on mitigating the adverse effects of OTA and enhancing growth efficiency, antioxidant capacity, liver and renal function, immune systems, and serum biochemical indicators in broilers.

## Materials and methods

### Production of ochratoxin A and *Spirulina*

The strain *Aspergillus ochraceus* (CGMCC 3.4412) was employed for the production of ochratoxin A. This particular strain was obtained from the Central Laboratory of Residues of Agricultural Products, located at the Agriculture Pesticides Residues Centre in Dokki, Egypt. The process of synthesizing OTA involved culturing the fungal strain in a liquid medium (containing 2% yeast extract and 20% sugar) for 8 days (12, 29). The concentration of ochratoxin A in the media was quantified using the stated technique in the AOAC (32) publication. The *Spirulina platensis* powder was purchased from Harraz Co, a local supplier based in Egypt.

### Housing and birds

The trial was carried out in the poultry farm at the Faculty of Veterinary Medicine, Nutrition and Clinical Nutrition Department, New Valley University, New Valley, Egypt. The facility, including drinkers and feeders, were cleaned and disinfected before the trial. The experimental conditions included maintaining a consistent temperature range of 22–25°C, controlling air humidity levels between 55 and 65%, and ensuring adequate ventilation. Broiler chicks (Ross 308) were 1 day old and showed similar mean body weights (40.82 ± 0.70 g) in all groups. Birds were obtained from a private hatchery and housed at a stocking density of 10 chicks in each cage. The experiment adheres to the regulations set by the New Valley University Ethics Committee for the utilization of experimental animals. Throughout the experiment, all groups had the same managerial and environmental conditions.

### Experimental design, diets, and treatments

The trial method used in this study was a randomized complete block design. One hundred sixty unsexed 1-day broiler chicks were assigned into four treatment groups, each contains four replicates of 10 chicks. The study lasted for 6 weeks (1–42 d). The experimental design is summarized in Table 1. All birds were reread under the same conditions and in clean environments and was provided with diets that were nutritionally balanced in order to supply their nutritional needs, as stated in NRC (33) (Table 2). Feed and drink were provided in *ad libtum*.

**Table 1:** Experimental design.

Groups	No. of birds	Tested materials^1^
G1	40	BD without supplementation
G2	40	1 mg OTA/kg diet
G3	40	1 g Sp/kg diet
G4	40	1 mg OTA +1 g Sp/kg diet

1 BD, Basal diet; OTA, Ochratoxin; Sp, *Spirulina platensis*.

**Table 2:** Composition and chemical analysis of the experimental diets (starter and finisher diets).

Items	Starter (1–21 d)	Finisher (22–42 d)
Ingredients (%)		
Yellow Corn	50.53	59.25
Soybean meal (44% CP)	38.50	33.50
Soybean oil	0.30	1.40
Bran	7.50	3.00
Mono Calcium phosphate	1.00	0.90
Limestone	1.30	1.20
Vit-min Premix^*^	0.30	0.30
NaCl	0.30	0.30
DL-Methionine	0.11	0.10
L-Lysine	0.11	0.01
Choline Chloride 60%	0.05	0.04
Total	100	100
Calculated analysis^**^ (%)		
Crude protein	23.00	20.00
Metabolizable Energy Kcal/kg diet	2,900	3,100
Calcium	1.00	0.90
Phosphorus (Available)	0.48	0.45
Lysine	1.40	1.20
Methionine + Cysteine	0.92	0.72
Crude Fibre	3.43	2.88
Linoleic acid	1.50	1.40

* Growth Vitamin and Mineral premix Each 2.5 kg consists of: Vit A 12,000,000 IU; Vit D3 2,000,000 IU; Vit. E. 10 g; Vit k3 2 g; Vit B1, 1,000 mg; Vit B2, 49 g; Vit B6, 105 g; Vit B12, 10 mg; Pantothenic acid, 10 g; Niacin, 20 g, Folic acid, 1,000 mg; Biotin, 50 g; Choline Chloride, 500 mg, Fe, 30 g; Mn, 40 g; Cu, 3 g; Co, 200 mg; Si, 100 mg and Zn, 45 g.

** Calculated according to NRC (33).

### Growth performance

All of the birds were measured for body weight (BW) at 0, 2, 4, and 6 weeks of age. Additionally, body weight gain (BWG) was measured throughout the duration of the experiment. In addition, the feed intake was constantly assessed in a replicated method throughout the study periods to calculate the feed conversion ratio (FCR = g of feed intake/g of body gain).

### Carcass traits

After the study, a total of 10 broiler chicks per group were selected randomly. These birds were subsequently weighed and manually slaughtered for the purpose of conducting carcass traits. The carcass weight, giblets, gizzard, heart, liver, and intestine were measured and stated as a percentage of the total weight at slaughter. Additionally, the dressing percentage was determined.

### Blood parameters

At the end of the study, birds were euthanized, and blood samples were collected using aseptic techniques into sterile tubes. The samples were let to coagulate and were then subjected to centrifugation at 4,000 rpm for a duration of 10 min. The obtained serums were stored at −20°C until they were prepared for examination. The spectrophotometric measurement of multiple parameters was performed using kits imported by Biodiagnostic Company (Giza, Egypt) and Mybiosource.com (San Diego, CA, USA). These parameters included the levels of total protein (Catalog No.: MBS165636), albumin (Catalog No.: MBS2881881), aspartate transaminase (AST) (Catalog No.: MBS740867), alanine transaminase (ALT) (Catalog No.: MBS266858), lactate dehydrogenase (LDH) (Catalog No.: MBS263022), triglycerides (Catalog No.: MBS1601908), total cholesterol (Catalog No.: MBS1601900), high-density lipoprotein (HDL) (Catalog No.: MBS040311), and low-density lipoprotein (LDL) (Catalog No.: MBS269281). The determination of serum globulin levels involved the subtraction of albumin levels from the total serum protein levels. The concentrations of immunoglobulins G (IgG), M [IgM], and A [IgA] were detected in plasma samples using available kits (Catalog No.: MBS260043, MBS706158 and MBS564152, respectively) provided by Mybiosource.com (San Diego, CA, USA). The activity of glutathione peroxidase (GPx) was detected in plasma by the kit available kits with catalog number MBS1604302 (Mybiosource.com, San Diego, CA, USA). The activity of superoxide dismutase (SOD), malondialdehyde (MDA), and total antioxidant capacity (TAC), was detected in plasma samples using available kits provided by Bio-diagnostic, Egypt, with the following catalog numbers (SD 25 21, MD 25 29, and TA 25 13, respectively) and a spectrophotometer manufactured by Shimadzu, Japan, in conjunction with other laboratory instruments according to Elbarbary et al. (34).

### Histopathological alteration

Bursa and thymus were extracted and stored in 10% neutral buffered formalin for 72 h for histological analysis. Following fixation, the samples underwent dehydration using increasing concentrations of ethanol, subsequently, the specimens were purified using xylene and subsequently encased in paraffin. Tissue sections measuring five micrometers in thickness were cut using a microtome. Hematoxylin and eosin (H&E) staining was applied to the slides and analyzed by a Leika DM500 light microscope to observe histological alterations (35).

### Immunohistochemical staining to PCNA

The immunohistochemistry technique used for proliferating cell nuclear antigen (PCNA) was performed according to (36). The sections of tissue underwent deparaffinization and rehydration. Following the wash with PBS (0.1 M, pH 7.2–7.4), the sections of tissue were exposed to a solution of 3.0% hydrogen peroxide in PBS at room temperature for 10 min. After being rinsed with PBS, the sections were treated with normal goat sera for 30 min to prevent nonspecific antibody binding. The sections were exposed to the rabbit anti-PCNA polyclonal antibody (bs-0754R, Bioss, Beijing, China) for 20 h at a temperature of 4°C. The antibody was diluted to a working concentration of 1:100. Following three consecutive washes in PBS, the sample was treated with a secondary antibody, specifically biotinylated goat anti-rabbit IgG, and then with streptavidin-biotin complex (SA1020, Boster, Wuhan, China). Afterward, the slices were gently stained with hematoxylin and immersed in 100% ethyl alcohol and xylene for 3 min before being covered with a coverslip. For the negative controls, the identical procedure was followed, with the exception that PBS was used instead of the primary antibody. The stained slices were photographed with a Leika DM500 digital camera.

### Statistical analysis

SAS (SAS Institute Inc., 2001) was used for the statistical analysis. A one-way ANOVA was utilized to analyze the performance, carcasses, serum components, and oxidative state using the post-hoc Newman–Keuls test (with the diet as the fixed factor). At *p* < 0.05, the significance was determined.

## Results

### Growth performance

The influence of dietary *Spirulina* supplement on body weight and body weight gain of broilers exposed to ochratoxin A illustrated in Table 3. The finding displayed a significant (*p* = 0.049) improvement in body weight and body weight gain at age 42 due to *Spirulina* supplementation, where G3 achieved the best results. While, ochratoxicosis significantly decreased body weight and body weight gain at the end of the experiment (42 days of age). Moreover, the influence of dietary *Spirulina* supplement on feed consumption and feed conversion ratio of birds exposed to ochratoxin A illustrated in Table 4. The result revealed a significant decline in feed intake in the ochratoxin-treated group (G2) (*p* = 0.001) during the (1–42) period. Furthermore, the result illustrated a significant improvement in FCR (*p* = 0.029) due to *Spirulina* supplementation during the period (1–42) and G3 revealed the best result (1.50).

**Table 3:** Effect of dietary *Spirulina* supplement on body weight and body weight gain of broilers exposed to ochratoxin A.

Parameter	Age (days)	Experimental groups				*p*-value
		^1^G1	^2^G2	^3^G3	^4^G4	
Body weight (g)	1	41.0 ± 0.58	41.0 ± 0.59	40.6 ± 0.088	40.7 ± 0.89	0.976
	14	416 ± 5.30	417 ± 5.80	420 ± 4.80	407 ± 6.10	0.241
	28	1,348 ± 16.9^a^	1,270 ± 18.4^b^	1,395 ± 17.6^a^	1,361 ± 14.9^a^	0.016
	42	2,193 ± 35.4^a^	2,051 ± 39.3^b^	2,536 ± 32.8^a^	2,218 ± 30.6^ab^	0.049
Body gain (g)	1–14	360 ± 6.9	361 ± 7.4	379 ± 5.5	366 ± 5.8	0.266
	15–28	946 ± 13.2	868 ± 17.2	979 ± 14.5	955 ± 18.1	0.070
	29–42	845 ± 21.5	781 ± 21.3	1,141 ± 23.2	857 ± 20.9	0.231
	1–42	2,152 ± 35.3^ab^	2,010 ± 38.6^b^	2,496 ± 35.4^a^	2,177 ± 31.5^ab^	0.049

^1^Control group; ^2^Ochratoxin-exposed group; ^3^*Spirulina*-supplemented group. ^4^*Spirulina* + Ochratoxin group; ^a,b,c^Means with different superscripts in each row are significantly different.

**Table 4:** Effect of dietary *Spirulina* supplement on feed intake and feed conversion ratio of broilers exposed to ochratoxin A.

Parameter	Age (days)	Experimental groups				*p*-value
		^1^G1	^2^G2	^3^G3	^4^G4	
Feed intake (g)	1–14	701 ± 6.3^a^	650 ± 5.9^c^	681 ± 6.1^ab^	665 ± 6.6^bc^	0.003
	15–28	1,586 ± 7.9^a^	1,441 ± 7.5^c^	1,491 ± 7.9^b^	1,506 ± 8.2^b^	0.001
	29–42	1,706 ± 13.4^a^	1,575 ± 12.9^bc^	1,555 ± 14.6^c^	1,590 ± 12.7	0.008
	1–42	4,010 ± 46.5^a^	3,661 ± 37.9^d^	3,710 ± 42.2^c^	3,756 ± 39.3^b^	0.001
Feed conversion ratio (g feed/g gain)	1–14	1.94 ± 0.03^a^	1.80 ± 0.04^b^	1.79 ± 0.02^b^	181 ± 0.04^b^	0.042
	15–28	1.68 ± 0.02^a^	1.67 ± 0.04^a^	1.53 ± 0.03^b^	1.60 ± 0.03^ab^	0.049
	29–42	2.03 ± 0.05	2.02 ± 0.07	1.46 ± 0.06	1.86 ± 0.07	0.092
	1–42	1.85 ± 0.05^a^	1.82 ± 0.06^a^	1.50 ± 0.05^b^	1.71 ± 0.07^ab^	0.029

^1^Control group; ^2^Ochratoxin-exposed group; ^3^*Spirulina*-supplemented group. ^4^*Spirulina* + Ochratoxin group; ^a,b,c^Means with different superscripts in each row are significantly different.

### Measurements of carcass

The effect of dietary *Spirulina* supplement on carcass traits of broilers exposed to ochratoxin A demonstrated in Table 5. The results illustrated a significant variation in carcass traits and the *Spirulina*-treated group presented a significant improvement (*p* = 0.048, *p* = 0.002) in carcass weight and dressing percentage (2,001 g, 79.2%). Moreover, revealed a significant decrease (*p* = 0.003) in abdominal fat percentage (1.27%). While, G2 revealed a significant reduction in carcass weight and dressing percentage (1,547 g, and 75.3%) respectively relative to control and other groups.

**Table 5:** Effect of dietary *Spirulina* supplement on carcass traits of broilers exposed to ochratoxin A.

Item	Experimental groups				*p*-value
	^1^G1	^2^G2	^3^G3	^4^G4	
Carcass weight (g)	1,698 ± 44.1^ab^	1,547 ± 41.6^b^	2,001 ± 42.2^a^	1,693 ± 39.5^ab^	0.048
Dressing %	77.1 ± 0.26^b^	75.3 ± 0.36^c^	79.2 ± 0.42^a^	76.2 ± 0.3^4bc^	0.002
Giblet %	4.70 ± 0.04	4.80 ± 0.06	4.70 ± 0.06	4.56 ± 0.05	0.595
Liver %	1.53 ± 0.03	1.54 ± 0.05	1.50 ± 0.06	1.52 ± 0.04	0.916
Gizzard %	2.43 ± 0.06	2.40 ± 0.08	2.46 ± 0.06	2.34 ± 0.05	0.736
Heart %	0.53 ± 0.02	0.56 ± 0.03	0.40 ± 0.04	0.50 ± 0.05	0.150
Abdominal fat %	1.57 ± 0.03^a^	1.53 ± 0.02^a^	1.27 ± 0.03^b^	1.60 ± 0.05^a^	0.003

^1^Control group; ^2^Ochratoxin-exposed group; ^3^*Spirulina*-supplemented group. ^4^*Spirulina* + Ochratoxin group; ^a,b,c^Means with different superscripts in each row are significantly differed.

### Serum biochemical parameters

The influence of *Spirulina* supplementation on the blood biochemical indices of broilers exposed to ochratoxin A was illustrated in Table 6. The result showed significant differences in liver and kidney function tests and G2 that was treated with Ochratoxin revealed a significant elevation (*p* = 0.001, *p* = 0.003) in liver enzymes ALT and AST (37.64 and 172.0 IU/L). Moreover, ochratoxicosis significantly increases (*p* = 0.001, *p* = 0.008) uric acid and creatinine levels (6.45 and 1.85 mg/dL, respectively) in the blood. G3 that was supplemented with *Spirulina* revealed a significant decrease (*p* = 0.001, *p* = 0.006, *p* = 0.008, *p* = 0.004) in cholesterol, triglycerides, LDL, and VLDL (178.2, 137.3, 71.17, and 34.69 mg/dL, respectively). While G2 that was treated with ochratoxin illustrated a significant elevation in cholesterol, triglycerides, LDL, and VLDL (357.8, 215.5, 124.7, and 65.43 mg/dL, respectively).

**Table 6:** Effect of dietary *Spirulina* supplement on blood biochemical indices of broilers exposed to ochratoxin A.

Item	Experimental groups				*p*-value
	^1^G1	^2^G2	^3^G3	^4^G4	
^5^ALT (IU/L)	24.66 ± 0.88^c^	37.64 ± 1.27^a^	24.0 ± 0.57^c^	32.33 ± 1.26^b^	0.001
^6^AST (IU/L)	147.8 ± 2.92^b^	172.0 ± 2.64^a^	144.3 ± 2.84^b^	162.0 ± 2.65^a^	0.003
^7^ALP (IU/L)	171.5 ± 2.20	171.6 ± 2.96	173.0 ± 2.66	172.6 ± 3.17	0.962
Uric acid (mg/dL)	4.05 ± 0.04^c^	6.45 ± 0.12^a^	4.02 ± 0.08^c^	5.02 ± 0.11^b^	0.001
Creatinine (mg/dL)	0.85 ± 0.07^c^	1.85 ± 0.06^a^	0.82 ± 0.05^c^	1.25 ± 0.06^b^	0.008
T. Cholesterol (mg/dL)	183.1 ± 5.9^c^	357.8 ± 13.9^a^	178.2 ± 5.3^c^	282.1 ± 2.7^b^	0.001
Triglycerides (mg/dL)	140.7 ± 4.85^c^	215.5 ± 5.53^a^	137.3 ± 4.38^c^	175.1 ± 5.12^b^	0.006
^8^HDL (mg/dL)	64.02 ± 3.82^ab^	49.02 ± 2.89^c^	69.67 ± 2.29^a^	59.48 ± 2.50^b^	0.008
^9^LDL (mg/dL)	74.84 ± 3.84^c^	124.7 ± 3.27^a^	71.17 ± 3.11^c^	103.2 ± 3.08^b^	0.001
^10^VLDL (mg/dL)	37.62 ± 2.48^c^	65.43 ± 3.62^a^	34.69 ± 2.14^c^	55.12 ± 2.95^b^	0.004

^1^Control group; ^2^Ochratoxin-exposed group; ^3^*Spirulina*-supplemented group. *^4^Spirulina* + Ochratoxin group; ^5^Alanine transaminase; ^6^Aspartate transferase; ^7^Alkaline phosphatase; ^8^High-density lipoprotein; ^9^Low-density lipoproteins; ^10^Very-low-density lipoproteins; ^a,b,c^Means with different superscripts in each row are significantly different.

### Antioxidant status

The influence of *Spirulina* supplementation on the antioxidant status of birds exposed to ochratoxin A was illustrated in Table 7. The finding presented significant variation in antioxidant activity and G3 that was supplemented with *spirulina* provided a significant decline (*p* = 0.001) in MDA (14.56 nmol/g) relative to the control. While G2 treated with ochratoxin illustrated the increased level of MDA (30.6 nmol/g). Moreover, G3 presented a significant increase (*p* = 0.015, *p* = 0.006, *p* = 0.036, *p* = 0.045) in SOD, GPx, CAT and TAC (439.5 u/g, 85.11 u/g, 38.96 nmol/g, and 141.2 nmol/g) respectively. Whereas the group supplemented with ochratoxin presented decreased levels of SOD, GPx, CAT, and TAC (279.4 u/g, 48.41 u/g, 25.20 nmol/g, and 108.1 nmol/g) respectively.

**Table 7:** Effect of dietary *Spirulina* supplement on antioxidant activity of broilers exposed to ochratoxin A.

Item	Experimental groups				*p*-value
	^1^G1	^2^G2	^3^G3	^4^G4	
MDA (nmol/g)	14.42 ± 0.71^c^	30.6 ± 0.87^a^	14.56 ± 0.63^c^	25.78 ± 0.93^b^	0.001
SOD (u/g)	374.0 ± 27.6^ab^	279.4 ± 323.7^b^	439.5 ± 23.4^a^	295.6 ± 28.7^b^	0.015
GPx (u/g)	81.35 ± 5.53^a^	48.41 ± 2.61^b^	85.11 ± 3.75^a^	58.82 ± 3.74^b^	0.006
CAT (nmol/g)	38.17 ± 3.17^a^	25.20 ± 3.02^b^	38.96 ± 2.72^a^	32.99 ± 2.89^ab^	0.036
TAC (nmol/g)	138.3 ± 9.55^a^	108.1 ± 7.15^b^	141.2 ± 7.80^a^	128.1 ± 6.91^ab^	0.045

^1^Control group; ^2^Ochratoxin-exposed group; ^3^*Spirulina*-supplemented group. ^4^*Spirulina* + Ochratoxin group; ^a,b,c^Means with different superscripts in each row are significantly different.

### Immunological indices

Effect of dietary *Spirulina* supplement on some immunological indices of broilers exposed to ochratoxin A illustrated in Table 8. The finding revealed a nonsignificant variation (*p* = 0.081) in phagocytic activity and group 3 supplemented with *Spirulina* showed an increased level of phagocytic activity (41.1) while group 2 supplemented with ochratoxin revealed decreased levels of phagocytic activity (24.94) relative to control and other treatments. Moreover, group 3 treated with *Spirulina* illustrated a significant (*p* = 0.003) elevation of the phagocytic index (0.50) while group 2 supplemented with ochratoxin revealed significantly decreased levels of the phagocytic index (0.20) relative to control and other treatments.

**Table 8:** Effect of dietary *Spirulina* supplement on some immunological indices of broilers exposed to ochratoxin A.

Item	Experimental groups				*p*-value
	^1^G1	^2^G2	^3^G3	^4^G4	
Phagocytic activity	34.99 ± 4.31	24.94 ± 3.21	41.11 ± 3.89	31.12 ± 2.88	0.081
Phagocytic index	0.37 ± 0.03^b^	0.20 ± 0.02^c^	0.50 ± 0.04^a^	0.28 ± 0.02^bc^	0.003

^1^Control group; ^2^Ochratoxin-exposed group; ^3^*Spirulina*-supplemented group. ^4^*Spirulina* + Ochratoxin group; ^a,b,c^Means with different superscripts in each row are significantly differed.

### Pathological and immunohistochemical staining

#### Bursa Fabricius

**Figure 1:**
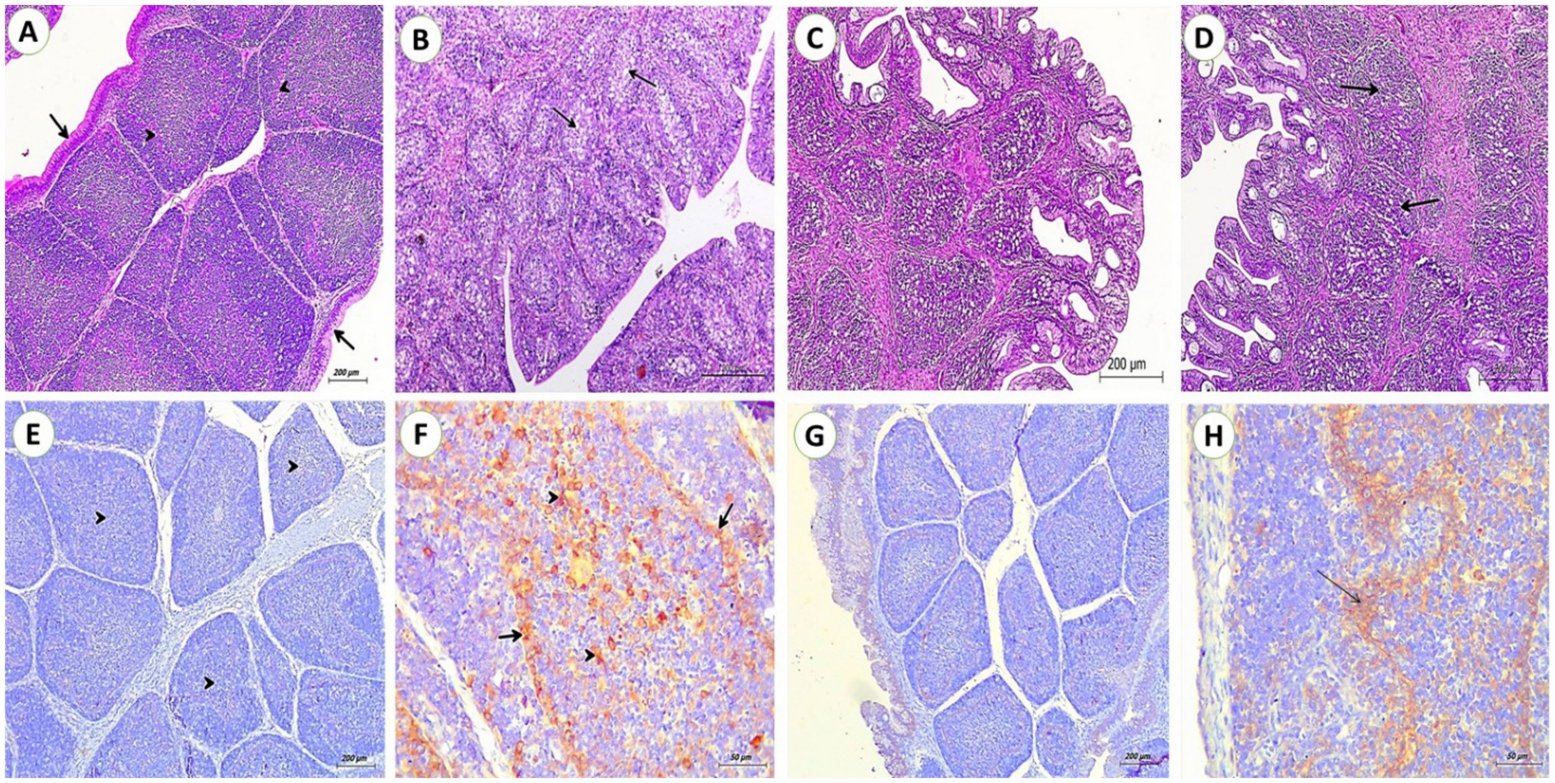
Bursa from broilers experimentally fed ochratoxin. **(A,C)** From control and SP groups showed folds Each fold contains tightly packed lymphoid follicles (arrow heads) separated by connective tissue and covered by pseudostratified columnar epithelium (arrow) (H&E, scale bar: 200). **(B)** From OTA group showed depletion of lymphoid follicles and extensive loss of the covering epithelium with exposing for the underlaying connective tissue. (H&E, scale bar: 200). **(D)** From OTA + SP group showed mild depletion of lymphoid follicles (H&E, scale bar: 200). **(E,G)** From control and SP groups showed no positive reaction for PCNA (IHC stain, scale bar: 200). **(F)** From OTA group showed a strong positive reaction for PCNA in lymphoid follicles (arrows heads) and follicle-associated epithelium (arrows) (IHC stain, scale bar: 50). **(H)** Mild positive reaction for PCNA in lymphoid follicles and follicle-associated epithelium of OTA + SP group (arrows) (IHC stain, scale bar: 50).

The control group showed normal folds each fold contains tightly packed lymphoid follicles (arrowheads) divided by connective tissue (CT) and covered by pseudostratified columnar epithelium. Compared to the control group, OTA fed group illustrated a loss of typical bursal architecture, cortical layer thinning, and medullary lymphoid depletion with lymphocytolysis. Some interfollicular edema was noticed. There was extensive loss of the covering epithelium with exposure to the underlying connective tissue. There were no alterations in SP fed group. Prominent amelioration of these changes in the OTA + SP group by the absence of edema and mild depletion of lymphoid follicles (Figures 1A–D). Regarding immunohistochemical reaction to PCNA, no reactions were detected in bursal tissues for the control and SP group. Strong positive reactions for PCNA in lymphoid follicles and follicle-associated epithelium were seen in the OTA group. Only mild reactions in lymphoid follicles and follicle-associated epithelium of the OTA + SP group were revealed (Figures 1E–H).

#### Thymus

**Figure 2:**
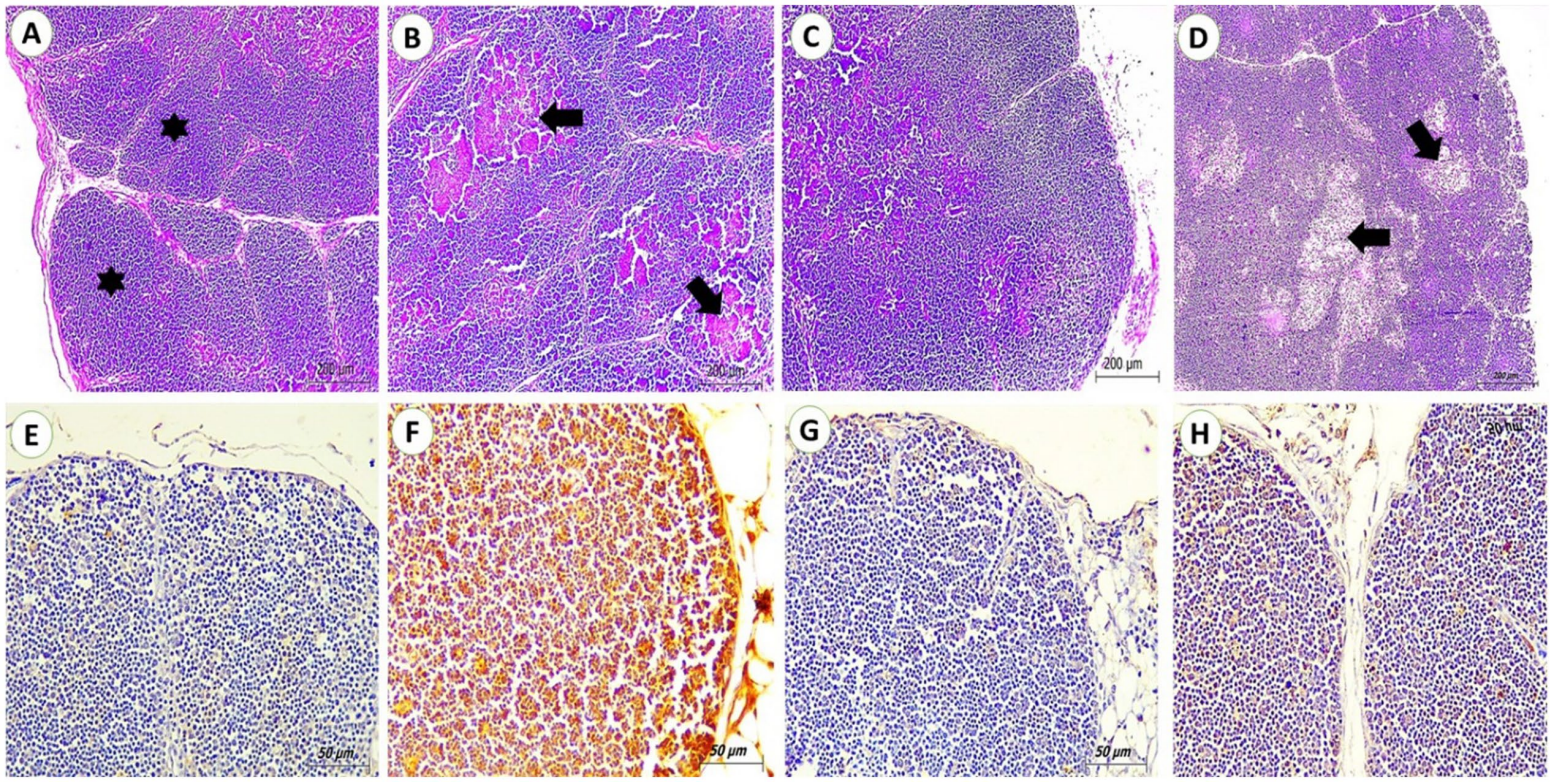
Thymus from broilers experimentally fed ochratoxin. **(A,C)** From control and SP groups showed connective tissue capsule and Septa from the capsule incompletely divide the cortex and end at the cortico-medullary junction with the dense lymphocyte population (stars) giving the cortex deep basophilic staining. (H&E, scale bar: 200). **(B)** From OTA group showed extensive hemorrhages and necrosis all over the cortex and medulla (arrows). (H&E, scale bar: 200). **(D)** From OTA + SP group showed focal depletion of lymphocyte populations (arrows) (H&E, scale bar: 200). **(E,G)** From control and SP groups showed no positive reaction for PCNA (IHC stain, scale bar: 50). **(F)** From OTA group showed a strong positive reaction for PCNA in lymphocyte populations (IHC stain, scale bar: 50). **(H)** Mild positive reaction for PCNA in lymphocyte populations of OTA + SP group (arrows) (IHC stain, scale bar: 50).

Both control and SP groups showed normal tissue architecture, connective tissue capsule, and septa partially separate the cortex, ending at the cortico-medullary junction. This area contains a high concentration of lymphocytes, resulting in a deep basophilic staining of the cortex. Relative to the control group, the OTA group showed severe tissue alterations; thymic lobular atrophy, nuclear fragmentation or even lysis, extensive hemorrhages, and necrosis all over the cortex and medulla. For the OTA + SP group, most microscopic changes were improved, only congestion and focal depletion of lymphocyte populations were detected (Figures 2A–D). Regarding immunohistochemical reaction to PCNA, no reactions were detected in thymus tissues for the control and SP group. A strong positive reaction for PCNA in lymphocyte populations was seen in the OTA group. Only mild reactions in lymphocyte populations of the OTA + SP group were detected (Figures 2E–H).

## Discussion

Research has shown that OTA poisoning can negatively impact the gastrointestinal system of hens, resulting in reduced food absorption and thus hindering their normal growth. Broiler hens that were given ochratoxin A (OTA) at concentrations of 20 or 50 μg/kg body weight (BW) experienced a reduction in both their overall body weight and weight gain (37, 38). The results of this study elaborated that supplementing *Spirulina* to the diet could successfully decrease the negative effect of OTA toxicity on broiler performance. The beneficial impacts of *Spirulina* are most likely due to its abundant concentration of antioxidants, vitamins, and other bioactive substances (39). These substances can counteract the harmful effects of oxidative stress and tissue damage produced by exposure to OTA. These data show that when chicks are fed a diet treated with up to 1 g/kg diet SP, their growth performance improves. These findings concur with those of Abou-Zeid et al. (40), who discovered that birds given a diet with 2 g of *Spirulina* per kg had superior body weight means. *Spirulina* enhances the assimilation of minerals, safeguards against diarrhea, and optimizes the process of nutrient digestion. The beneficial effects of adding *Spirulina* to the diet of broiler chickens can be recognized due to its high levels of amino acid digestibility and metabolizable energy (39). This is particularly important considering the negative effect of heat stress on the structure of the intestines and the amount of feed consumed (41).

The findings of our research revealed that supplementing *Spirulina* in the diet might effectively alleviate the negative impacts of OTA stress on broiler carcass characteristics. The findings of this trail align with the findings of Abou-Zeid et al. (40), who found that birds who were given a meal containing 2 g of *Spirulina* per kg showed a notable variation in carcass and abdominal fat %. However, there was no significant variation in liver, heart, or gizzard percentage across the different groups.

The outcomes of this trial indicate that adding *Spirulina* to the diet can successfully mitigate the adverse impacts of OTA stress on blood parameters, and antioxidant levels. Consistent with the findings of the current investigation by Pestana et al. (42), there was a significant elevation in total protein concentration (*p* ≤ 0.001) relative to the other groups of birds. The improved digestibility of protein seen in diets supplemented with *Spirulina* may be attributed to enhanced absorption, leading to increased development in broiler chickens (23). These findings agree with the results of former studies applied by Fathi (43) and Opoola et al. (44). They observed that chickens receiving diets containing *Spirulina* at doses of 6, 12, and 18 g/kg had significantly increased levels of globulin, glucose, and total protein relative to those on a control diet.

The scientists proposed that the elevated levels of serum protein, globulin, and albumin could be attributed to the superior protein content and the amount of *Spirulina platensis*, which is abundant in phycocyanin and polyunsaturated fatty acids (45). The plasma lipids profile revealed that the levels of plasma cholesterol and total lipids were reduced in all supplemented groups relative to the control group. Abdel-Hady and EI-Ghalid (46) noticed similar findings when they discovered that treating a broiler diet with 3 and 6% *Spirulina* led to a substantial reduction in serum concentration of total lipid, triglyceride, cholesterol, and low-density lipoprotein in both experimental groups.

The inclusion of *Spirulina* in the diet of birds resulted in a reduction in their blood lipid profile. The concentration of high-density lipoprotein (HDL) in broilers increased significantly (*p* ≤ 0.05) when they were given 1 g of *Spirulina*. *Spirulina* has been found to reduce cholesterol concentration in the blood by affecting the metabolism of lipoproteins and increasing the activity levels of lipoprotein enzymes. This hypocholesterolemic effect is achieved by lowering both plasma and liver cholesterol levels through the increased action of lipoprotein lipase and hepatic triglyceride lipase (45). The liver is considered as the primary metabolic organ in the body (47). It demonstrates the hepatoprotective impacts of *Spirulina*, which can be attributed to its antioxidative and anti-inflammatory properties (48). The concentration of AST and ALT showed a significant decrease in the *Spirulina* groups. These results align with the results reported by Abaza et al. (49) and Jamil et al. (48), they showed that the activity of ALT and AST decreased significantly in all treatment groups that were supplemented with *Spirulina*. Similarly, Zeweil et al. (50) found that supplementing chickens with *Spirulina* at levels of 0.5 and 1 g/kg in their diet decreased the adverse impacts of heat stress on ALT and AST levels. There was also a slight reduction in plasma ALP and activities of liver enzymes, but these levels stayed within the normal range observed in the present study, suggesting normal liver function. The supplemented groups exhibited a reduction in ALP, ALT, and AST concentration. The finding by Abdel-Daim et al. (51) illustrated that *Spirulina* has a hepatoprotective effect, which can restore the normal concentration of liver enzymes and improve liver health. The rise in serum ALP, ALT, and AST activity has been linked to physiological stressful conditions (52) such as ochratoxicosis. This contradicts the results of Sugiharto et al. (53), who revealed that administering supplements containing 1% *Spirulina platensis* for seven, 21, and 35 days had no significant influence on AST and ALT levels. The conflicting outcomes of the research may be attributed, to some part, to the variation in the nutritional and functional characteristics of the *Spirulina platensis* utilized. Moreover, *Spirulina* supplementation decreased the adverse effect of OTA on the kidney function tests and decreased the uric acid and creatinine in the blood relative to other groups and controls. on the other hand, our findings agree with Li et al. (54) who applied a study to investigate the influence of adding OTA to the feed of birds at a concentration of 50 g/kg. They noticed that the presence of OTA resulted in elevated MDA concentration in the kidneys, whereas the TAC was decreased. In addition, the concentration of SOD, CAT, and GPx was significantly reduced. The results indicate that OTA induces the production of reactive oxygen species, resulting in oxidative stress in the kidneys of birds (55).

The findings demonstrated that the addition of *Spirulina* effectively alleviated the adverse effects of ochratoxin on some immunological parameters in broiler chickens, and boosted phagocytic activity and phagocytic index relative to control and other groups. Our results align with (25, 56, 57) who reported that *Spirulina* has demonstrated a distinct effect on monocytes and natural killer (NK) cells, which are vital constituents of the innate immune system. The supplementation of *Spirulina* has been shown to improve the phagocytic response of macrophages and the action of natural killer (NK) cells in both chickens and humans.

The results of this study illustrated that supplementing *Spirulina* to the broiler’s diet can successfully mitigate the adverse effect of OTA toxicosis on broiler pathological changes of lymphoid organs such as the bursa and thymus. Lymphocyte depletion, necrosis, and hemorrhage were observed in the OTA group. Chickens with underdeveloped bursal follicles are known to have heightened susceptibility to bacterial (11, 58) and viral (59) infections. Because lymphoid organs have an important role in humoral immunological responses. Therefore, these histological abnormalities have the potential to negatively impact the humoral immune function in hens following exposure to OTA. Our finding illustrated that OTA caused the rise of PCNA proteins in the bursa and thymus, that essential for replication. It acts as a scaffold to recruit proteins involved in DNA replications. Since PCNA requires ubiquitination to carry out its biological role, the increase in PCNA levels cannot definitively be attributed to its enhanced functionalities (36).

## Conclusion

In conclusion, the administration of *Spirulina* (1 g/kg diet) in broiler chickens fed on ochratoxin A (1 mg/kg diet) contaminated diet or as a standalone supplement illustrated a decrease in the adverse impacts of ochratoxin A, additionally improving the growth performance, antioxidant activity, liver and kidney function, immune response, of broiler chicken. Using *Spirulina* up to 1 g/kg of diet can be beneficial and a good strategy in improving health, performance and solving the OTA problem in poultry farms. From our results, the future of *Spirulina* as a functional supplement looks promising due to its good benefits. However, before this novel additive can be widely used in poultry diets, large-scale commercial production of *Spirulina* for the feed sector must be established.

## Data Availability

The raw data supporting the conclusions of this article will be made available by the authors without undue reservation.
